# Current and Prospective Targets of Pharmacologic Treatment of Hereditary Angioedema Types 1 and 2

**DOI:** 10.1007/s12016-021-08832-x

**Published:** 2021-01-09

**Authors:** Lauré M. Fijen, Konrad Bork, Danny M. Cohn

**Affiliations:** 1 Department of Vascular Medicine, Amsterdam UMC, University of Amsterdam, Amsterdam, Netherlands; 2 Department of Dermatology, University Medical Center, Johannes Gutenberg University, Mainz, Germany

**Keywords:** Hereditary angioedema, C1-inhibitor, Bradykinin, Contact activation system, Kallikrein/kinin system, Serine protease

## Abstract

Hereditary angioedema (HAE) is a rare disease that causes episodic attacks of subcutaneous and submucosal edema, which can be painful, incapacitating, and potentially fatal. These attacks are mediated by excessive bradykinin production, as a result of uncontrolled activation of the plasma kallikrein/kinin system, which is caused by a C1 esterase inhibitor deficiency or dysfunction in HAE types 1 and 2, respectively. For many years, treatment options were limited to therapies with substantial adverse effects, insufficient efficacy, or difficult routes of administration. Increased insights in the pathophysiology of HAE have paved the way for the development of new therapies with fewer side effects. In the last two decades, several targeted novel therapeutic strategies for HAE have been developed, for both long-term prophylaxis and on demand treatment of acute attacks. This article reviews the advances in the development of more effective and convenient treatment options for HAE and their anticipated effects on morbidity, mortality, and quality of life. The emergence of these improved treatment options will presumably change current HAE guidelines, but adherence to these recommendations may become restricted by high treatment costs. It will therefore be essential to determine the indications and identify the patients that will benefit most from these newest treatment generations. Ultimately, current preclinical research into gene therapies may eventually lead the way towards curative treatment options for HAE. In conclusion, an increasing shift towards the use of highly effective long-term prophylaxis is anticipated, which should drastically abate the burden on patients with hereditary angioedema.

## Introduction

Hereditary angioedema (HAE) is a rare, disabling disorder, with symptoms ranging from disfiguring and incapacitating peripheral swellings, painful abdominal episodes, to potentially fatal laryngeal or oropharyngeal edema [1]. Before the availability of medication and adequate airway support, HAE-related mortality was estimated to be as high as 33 to 40% as a result of asphyxiation [2, 3]. For many years, treatment options were limited to fresh frozen plasma (FFP) infusions, antifibrinolytics, progestins, and attenuated androgens (AA). In the past decade various new targeted HAE therapeutics have been developed, with an emphasis on safety, efficacy, and easier routes of administration (including enhanced options for self-administration). Following a brief overview of the pathophysiology of HAE, this paper will review all currently available therapies and new treatment approaches in development for HAE with C1 esterase inhibitor (C1-INH) deficiency.

### Pathophysiology

HAE is an autosomal dominant inherited disorder. Mutations in *SERPING1*, the gene that encodes the serine protease inhibitor (SERPIN) C1-INH, account for HAE with C1-INH deficiency (C1-INH-HAE). The two types of C1-INH-HAE, type 1 and type 2, are clinically indistinguishable but are caused by different mutations in the *SERPING1* gene. In C1-INH-HAE type 1, the mutations result in truncated or misfolded proteins that are incompletely secreted, leading to decreased plasma levels of C1-INH which are less than 50%, generally 5 to 30%, of normal plasma levels. Mutations causing C1-INH-HAE type 2 are located at exon 8 or near the active site, leading to a fully secreted, but dysfunctional C1-INH protein. Hence, C1-INH functional levels in plasma are decreased in both types, whereas C1-INH antigenic levels are only decreased in C1-INH-HAE type 1 [4]. Other, even more rare, types of HAE are not associated with C1-INH deficiency (HAE with normal C1-INH; nC1-INH-HAE, formerly referred to as HAE type 3) and include those associated with mutations in the genes expressing factor XII [5], plasminogen [6], angiopoietin-1 [7], kininogen-1 [8], and myoferlin [9]. In one or more remaining types of nC1-INH-HAE, the genetic cause is still unknown. This review will focus on C1-INH-HAE types 1 and 2 as the clinical trials for new HAE therapies are predominantly focusing on these specific types.

C1-INH is a heavily glycosylated single chain SERPIN of 478 amino acid residues. It is the main inhibitor of various complement proteases (C1r, C1s, and mannose-binding lectin-associated serine protease; MASP 1 and 2), and contact system proteases (activated factor XII; FXIIa and plasma kallikrein; PKa). In addition, C1-INH is an efficient inhibitor of the intrinsic coagulation pathway (inhibition of activated factors XII and XI) and the fibrinolytic pathway (inhibition of plasmin). If C1-INH levels are decreased or the protein is defective, the kallikrein/kinin system (KKS) is inadequately controlled, leading to excessive release of the nonapeptide bradykinin (BK) which is the predominant mediator of enhanced vascular permeability in angioedema attacks [10, 11].

**Fig. 1:**
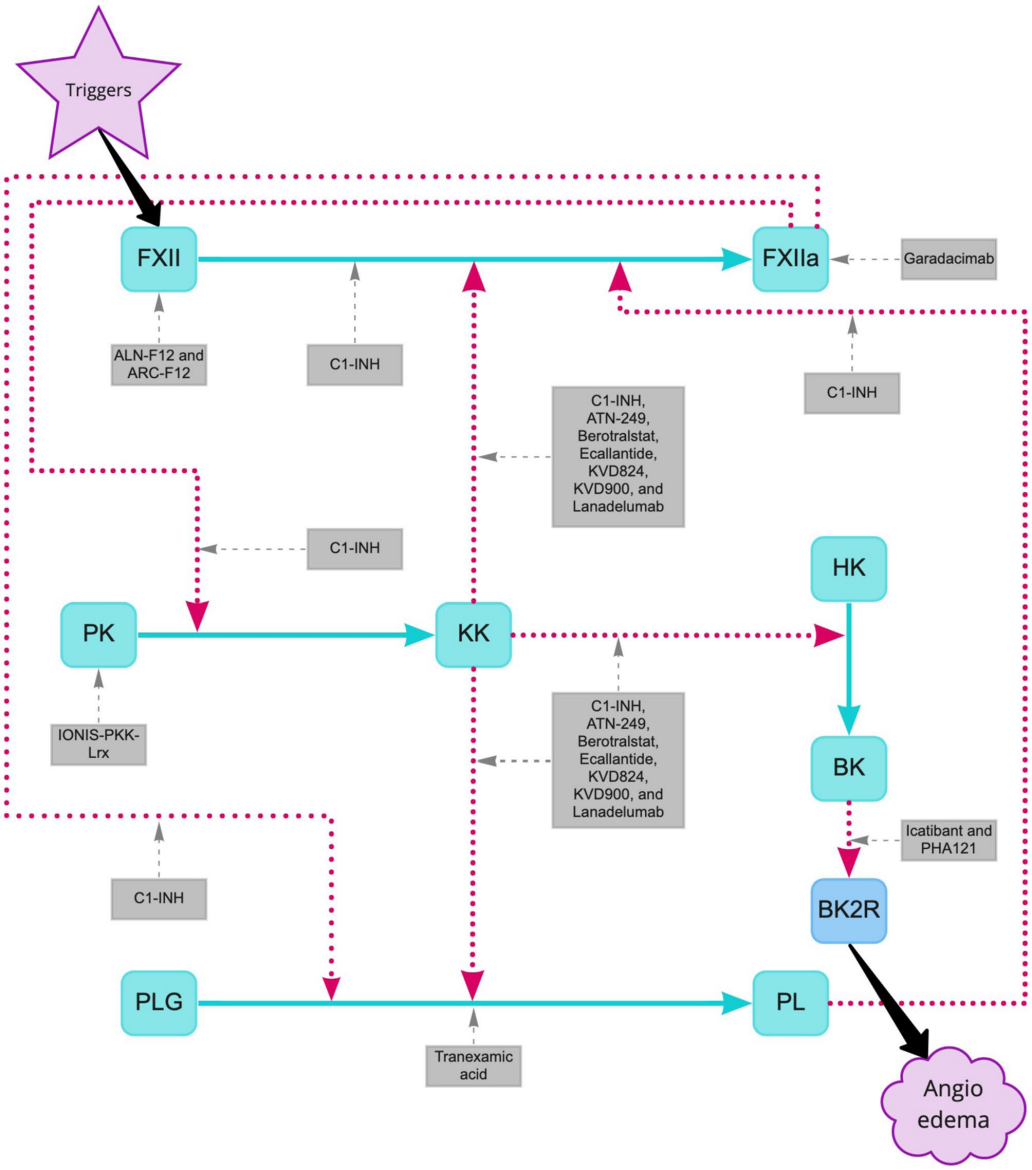
Pathways inhibited by current HAE drugs and developmental treatment options. Activation of the contact system is initiated by activation of Factor XII (FXII), resulting in activated Factor XII (FXIIa). FXIIa can activate prekallikrein (PK) to plasma kallikrein (PKa), which in turn can activate FXII to produce more FXIIa. In addition, PKa cleaves bradykinin (BK) from high-molecular-weight kininogen (HK). Finally, BK ligates the bradykinin B2 receptor (BKRB2). Additionally, FXIIa and PKa can activate plasmin (PL) from plasminogen (PLG). PL further stimulates the activation of FXII. Activation is indicated by red big-dotted arrows and inhibition by HAE drugs by gray small-dotted arrows*.* C1-INH denotes C1 esterase inhibitor

During attacks of C1-INH-HAE, activation of the contact system is initiated by autoactivation of factor XII (FXII), resulting in FXIIa. If uncontrolled by C1-INH FXIIa can activate prekallikrein (PK) to the proteolytic enzyme PKa, which in turn cleaves FXII to produce more FXIIa (see Fig. 1) In addition, the activated PKa liberates BK from high-molecular-weight kininogen (HK) [12, 13]. Once BK ligates the BK B2 receptor (BKRB2) which is constitutively expressed on endothelial cells, nitric oxide is produced leading to simultaneous smooth muscle cell relaxation and transmembrane vascular endothelial cadherin molecule degradation, causing vascular leakage between the endothelial cells [14, 15]. Moreover, FXIIa, PKa, and BK can activate plasmin from its zymogen plasminogen, thereby activating the fibrinolytic pathway. Plasmin is pivotal for the degradation of cross-linked fibrin. Therefore, plasmin activation results in increased fibrin degradation products including D-dimers that may be increased even in asymptomatic HAE patients. Indeed, D-dimer levels further increase during acute attacks [16]. Plasmin also stimulates the activation of FXII via positive feedback [17]. FXIIa in turn can activate factor XI, thereby initiating the intrinsic coagulation cascade. Nevertheless, HAE is not associated with an increased thrombotic risk.

### Historical and Current Prophylactic Treatment

Treatment of HAE can be divided into two strategies: (1) treatment of acute attacks (on demand therapy; ODT) and (2) prevention of attacks, which includes short-term prophylaxis (STP) and long-term prophylaxis (LTP). It is notable that in the past the decision to initiate LTP used to be solely based on attack frequency, whereas it is nowadays very well recognized that not only burden of disease but also patient preferences should be taken into account [18]. STP is indicated before all procedures that can induce an attack, such as (dental) surgery, endotracheal intubation and endoscopy.

In contrast to angioedema with urticaria, which is predominantly histamine-mediated, antihistamines, corticosteroids, and epinephrine are ineffective in HAE [19]. For LTP, *progestins* were occasionally used to inhibit the effects of *estrogens* which may increase FXII, PKa, and HK levels, resulting in (over) consumption of C1-INH [20]. *Progestins* were reported to have a positive effect on the frequency of angioedema attacks in women with HAE. In most women they are well tolerated, but *progestins* carry the potential to cause breakthrough vaginal bleeding, pelvic discomfort, and mastalgia [21].

Until the first administration of *intravenous plasma-derived *C1-INH* concentrate* in 1979, and its Food and Drug Administration (FDA) approval in 2008, the only recommended options for LTP were *attenuated androgens* (AA) and *antifibrinolytics* [22, 23].

AA, such as *danazol*, *oxandrolone*, and *stanozolol*, are administered orally. Their mode of action is through induction of C1-INH synthesis in hepatocytes [24] and increased expression of C1-INH mRNA in peripheral blood mononuclear cells [25]. Furthermore, AA induces aminopeptidase P activity, which catabolizes BK [26]. Although AA are effective in a considerable proportion of HAE patients, their long-term use is limited due to a number of potential adverse effects [27]. AA are contraindicated in children and pregnant women (because of their virilising effects), as well as in patients with liver diseases, breast cancer, and prostate cancer [28]. In addition, AA use has been linked to increased cardiovascular risk and hepatocellular carcinoma. To minimize adverse effects, the optimal dose for *danazol* is ≤ 200 mg per day. AA has been recommended for STP in the past, but C1-INH* concentrates* are currently considered the pre-procedural prophylaxis of choice. Very frequent use of AA for STP may lead to similar side effects as seen with long-term use. Nevertheless, STP with AA is considered to be safe, even in children. AA are used for five days before until two to three days after the procedure [18] (see Table 1).

**Table 1:** Current treatment options for hereditary angioedema with C1-inhibitor deficiency

Drug (trade name)	Manufacturer	Mechanism of action	Indication	Administration	Age indications
Attenuated androgens: danazol, oxandrolone, and stanozolol	Generic manufacturers	AA induce aminopeptidase P activity and increase C1-INH synthesis and C1-INH mRNA expression	LTP, STP	Oral	≥18 years
Berotralstat (Orladeyo®)	BioCryst Pharmaceuticals	Kallikrein inhibitor	LTP	Oral	FDA:≥12 years, under review for EMA approval
Plasma-derived C1-INH (Berinert®)	CSL Behring	Plasma-derived C1-INH concentrate	ODT, STP	Intravenous	All
Plasma-derived C1-INH (Cinryze®)	Takeda	Plasma-derived C1-INH concentrate	LTP, ODT, STP	Intravenous	LTP ≥6 years, ODT and STP ≥2 years
Conestat alfa (Ruconest®)	Pharming Group NV	Recombinant C1-INH concentrate	ODT	Intravenous	FDA: adolescents and adults EMA: ≥2 years
Ecallantide (Kalbitor®)	Takeda	Kallikrein inhibitor	ODT	Subcutaneous, no self-administration	≥12 years
Plasma-derived C1-INH (Haegarda®)	CSL Behring	Plasma-derived C1-INH concentrate	LTP	Subcutaneous	≥12 years
Icatibant (Firazyr®)	Takeda	BKRB2 antagonist	ODT	Subcutaneous	FDA: ≥18 years EMA: ≥2 years
Lanadelumab (Takhzyro®)	Takeda	Kallikrein inhibitor	LTP	Subcutaneous	≥12 years
Tranexamic acid (Cyklokapron®)	Generic manufacturers	Competitive inhibitor of plasminogen-mediated FXII activation	LTP	Oral	Adolescents and adults

*AA* attenuated androgens, *BKRB2* bradykinin receptor B2, *C1-INH* C1 esterase inhibitor, *LTP* long-term prophylaxis, *ODT* on demand treatment, *STP* short-term prophylaxis

*Tranexamic acid* is an orally available antifibrinolytic agent. The recommended dosage is 30–50 mg/kg daily, divided in two or three doses to a maximum of six grams per day. This lysine analogue holds a wide range of SERPIN activity, amongst which is the competitive inhibition of plasminogen. Thus, the activation of plasminogen into plasmin is reduced, thereby preventing plasminogen-mediated amplification of FXII activation. In most C1-INH-HAE patients, treatment with *tranexamic acid* is not sufficiently effective. Current international guidelines on HAE do recommend neither AA nor *antifibrinolytics* as a first-line therapy [29].

C1-INH* concentrates* supplement the deficient or replace the dysfunctional C1-INH in HAE patients, thus enabling inhibition of FXIIa and PKa in order to prevent excessive BK production. *Plasma-derived *C1-INH* concentrates* (*Cinryze®* and *Berinert®*) are an effective option for both LTP and STP [30–32]. The recommended dosages for STP with *plasma-derived *C1-INH *concentrates* are 1000 IU or 20 IU/kg, whereas the recommended LTP dose for *Cinryze®* is 1000 IU per 3–4 days. *Berinert*® has not been licensed for LTP. Although intravenous access is required for administration, many patients can be trained for self-administration which was found to be safe and highly effective [33]. It is of note however that long-term venous access may become complicated. Indwelling central catheters have been inserted in a subgroup of these patients, thereby risking serious complications such as thrombotic events and infections [34].

*Haegarda*® is a new generation of *plasma-derived *C1-INH* concentrate* that is administered subcutaneously. It was licensed in 2017 by the FDA for LTP in adolescents and adults. Clinical efficacy was demonstrated in the phase 3 Clinical Studies for Optimal Management in Preventing Angioedema with low-volume subcutaneous C1-inhibitor replacement Therapy (COMPACT) trial, in which a total of 83% of patients receiving the recommended dose of self-administered 60 IU/kg twice weekly were free of attacks in the long-term extension arm [35]. Moreover, this treatment improved HAE-related quality of life (QoL) [36].

*Lanadelumab* (*Takhzyro*®) is another subcutaneously administered drug that has been licensed for LTP in patients aged 12 years and older. This fully human IgG1 monoclonal antibody directed against PKa was approved by the FDA in 2018. In the phase 3 Hereditary Angioedema Long-term Prophylaxis (HELP) study, the average breakthrough attack rate was significantly lower in the groups that received 300 mg every 2 weeks and every 4 weeks as compared with placebo (respectively 0.26 and 0.53 versus 1.97 attacks per month) [37]. In addition, 66.7% of patients receiving 300 mg fortnightly reported a reduction of ≥ 90% of angioedema attacks during the treatment course of 26 weeks. Achievement of the minimal clinically important difference in total QoL-score was reported across all treatment arms (including 37% receiving placebo), with the greatest improvement (81%) shown in the 300 mg every 2 weeks arm which is the currently recommended dose. A tentative prolongation of the dosing interval to once every 4 weeks can be considered once clinical remission is achieved.

The most recent addition to the arsenal of LTP is the oral PKa inhibitor *berotralstat* (*Orladeyo*®, formerly BCX7353), approved for HAE patients aged 12 years and older by the FDA. The Efficacy and Safety Study of BCX7353 as an Oral Treatment for the Prevention of Attacks in HAE (APeX-2) study, a phase 3, randomized, double-blind, placebo-controlled study showed a 44% reduction in attack rate compared with placebo [38]. Notably, gastrointestinal side effects occurred frequently across all three study arms (42% in the group that received 110 mg, 50% in the 150 mg arm, and up to 36% in the placebo group) [39]. An open-label long-term safety study assessing the dose of 125 mg once daily is currently being conducted and an application for EMA approval has been submitted [40].

### Historical and Current on Demand (acute) Treatment

Historically, HAE patients were treated with high volumes of *fresh-frozen plasma *(FFP) for acute attacks, because FFP are easily available and assumed to contain some amount of functional C1-INH from the donor’s plasma [41, 42]. Although its benefit has never been objectively established in randomized controlled trials, FFP might be effective provided that large volumes are used. However, FFP bears the risks of transmission of blood borne pathogens and could theoretically exacerbate an HAE attack or escalate its severity, due to plasma kininogens that may provide substrate for additional BK release [43]. Therefore, FFP is only recommended for life-threatening attacks when other ODT options are lacking [29].

*Plasma-derived *C1-INH* concentrates* (*Cinryze*® and *Berinert*®) are effective and recommended options for ODT, using 1000 IU of *Cinryze*® or 20 IU/kg of *Berinert*® in children and adults. Although the use of other plasma-derived products (e.g., clotting factor concentrates) has led to the transmission of blood borne viral infections such as human immunodeficiency virus and hepatitis C, the transmission risk has almost completely abated for C1-INH* concentrates* since the application of nanofiltration. This technique prevents enveloped and non-enveloped viruses and likely even prions from contaminating these plasma products.

In 2017, Shire Pharmaceuticals reported a supply shortage of *Cinryze*® in many European Union countries and the United States of America (USA) as the demand had exceeded the production capacity. Such shortages can be circumvented with the use of *recombinant *C1-INH* concentrate conestat alfa* (*Ruconest*®) which does not depend on plasma donors and can be produced in large quantities. *Conestat alfa* is isolated from the milk of transgenic rabbits harbouring genomic human C1-INH sequences. Its glycosylation pattern differs from *plasma-derived *C1-INH* concentrate*, resulting in a lower half-life, due to glycosylation-dependent clearance of C1-INH concentrate in the liver [44]. Currently, *conestat alfa* is only licensed for ODT, dosed at 50 IU/kg in patients < 84 kg or 4200 IU in patients with a body weight of ≥ 84 kg. Despite its lower half-life, *conestat alfa* has also shown efficacy for STP [45], as well as for LTP [46]. These findings suggest that the treatment effect does not solely depend on plasma concentrations of C1-INH. Theoretically, *recombinant *C1-INH may be bound to endothelial cells and still be biologically active though undetectable in plasma.

To date, *ecallantide* (*Kalbitor*®, formerly DX-88) is the only direct PKa inhibitor available for ODT in patients aged 12 years and older (USA only), with a recommended subcutaneous dosage of 30 mg [47]. In the Evaluation of DX-88′s Effects in Mitigating Angioedema (EDEMA) 4 trial, a phase 3, double-blind, placebo-controlled randomized trial, *ecallantide* showed a significant reduction in mean symptom complex severity score and was associated with a significantly larger mean treatment outcome score compared with placebo [48]. Since anaphylaxis was reported in 3.5% of patients, *ecallantide* contains a black box warning in the USA and may only be administered by a healthcare professional in a setting with adequate medical support [49, 50].

More downstream inhibition of the KKS and the effects of BK on the vessel wall can be achieved with *icatibant* (*Firazyr*®), a synthetic decapeptide which acts as a competitive selective antagonist of the BK2 receptor. The For Angioedema Subcutaneous Treatment (FAST)-3 trial, a randomized, double-blind, placebo-controlled phase 3 study demonstrated a significantly shorter time to onset of symptom relief with *icatibant* (2.0 h) than with placebo (19.8; *P* < 0.0001) [51]. An important observation in further observational studies was the faster resolution of an angioedema attack when *icatibant* was administered early compared with late treatment [52]. *Icatibant* can be self-administered subcutaneously at a 30-mg dose and is licensed for ODT in patients aged 2 years and older by the European Medicines Agency (EMA), although the FDA approved *icatibant* only in adults.

### Future HAE Treatment Strategies

As long as absolute curation of HAE remains impossible, the development of newer drugs is focusing on increased efficacy, tolerability, and enhanced ease of administration. The newest LTP HAE drugs currently under development are highly specific (targeting a single protein of the KKS or contact system) and are either administered less frequent (once monthly or less) or orally. Likewise, some of the newest ODT drugs in clinical development are administered orally.

Several LTP therapies currently in development are specifically targeting FXII(a), the initiator of the BK-producing pathway. *Garadacimab*® (formerly CSL312) is the first fully human monoclonal antibody directed against FXIIa. The first clinical trial was initiated in 2017 and analysis of the first randomization period of the phase 2 trial showed an almost 99% reduction in breakthrough attacks compared with placebo, with a favourable safety profile [53] (see Table 2). Another mode of FXII inhibition is through *small interfering RNA (siRNA),* a sophisticated technique in which intracellular translation of FXII mRNA into the FXII protein is prevented by ~ 20–30 nucleotide RNA molecules. Two *siRNAs* are in preclinical development: *ALN-F12*® and *ARC-F12*® [54, 55]. Treatment with *siRNAs* seems promising, as the very long half-lives allow dosing as infrequent as twice annually [56].

**Table 2:** Developmental treatments for hereditary angioedema with C1-inhibitor deficiency

Drug (trade name)	Manufacturer	Mechanism of action	Planned indication	Administration	Regulatory status
ALN-F12®	Alnylam Pharmaceuticals	RNA interference targeted at FXII	LTP	Subcutaneous	Preclinical development
ARC-F12®	Arrowhead Pharmaceuticals	RNA interference targeted at FXII	LTP	Subcutaneous	Preclinical development
ATN-249®	Attune Pharmaceuticals	Kallikrein inhibitor	LTP	Oral	Phase 1 trial is complete
Berotralstat (BCX7353)	BioCryst Pharmaceuticals	Kallikrein inhibitor	ODT	Oral	Phase 2 trial is complete
BMN 331®	BioMarin	Adeno-associated virus-mediated antibody delivery gene therapy	LTP	Intravenous	Preclinical development
Conestat alfa (Ruconest®)	Pharming Group NV	Recombinant C1-INH concentrate	LTP	Intravenous	Phase 2 trial is complete
Garadacimab®	CSL Behring	Humanised anti-FXIIa monoclonal antibody	LTP	Subcutaneous	Phase 2 trial is recruiting
IONIS-PKK-L_Rx_®	IONIS Pharmaceuticals	Antisense oligonucleotide targeted at prekallikrein	LTP	Subcutaneous	Phase 2 results are expected Q2 2021
KVD824®	KalVista Pharmaceuticals	Kallikrein inhibitor	LTP	Oral	Phase 2 trial is expected to start in 2021
KVD900®	KalVista Pharmaceuticals	Kallikrein inhibitor	ODT	Oral	Phase 2 trial data are expected in Q1 2021
NTLA-2002®	Intellia Therapeutics	CRISPR/Cas9 editing of KLKB1	LTP	Intravenous	Preclinical development
PHA022121®	Pharvaris	BKRB2 antagonist	LTP, ODT	Oral	Phase 2 trial is in progress

*BKRB2* bradykinin receptor B2, *C1-INH* C1 esterase inhibitor, *LTP* long-term prophylaxis, *ODT* on demand treatment

More downstream inhibition of the KKS is accomplished by targeting plasma PK. *IONIS-PKK-L*_*Rx*_® is a second-generation *antisense oligonucleotide* that can selectively reduce hepatic plasma prekallikrein mRNA synthesis. The phase 1 study with *IONIS-PKK-L*_*Rx*_® showed a well-tolerated safety profile with reductions in plasma prekallikrein up to 94%. In addition, two patients with severe BK-mediated angioedema, including one patient with C1-INH-HAE, have been treated with *IONIS-PKK-L*_*Rx*_® under a compassionate use protocol and their attack rate was effectively reduced, with a good tolerability of the drug [57]. A phase 2 trial comparing subcutaneous *IONIS-PKK-L*_*Rx*_® 80 mg once monthly to placebo is ongoing [58]. Another investigational preparation targeting prekallikrein is *NTLA-2002*®, which is in preclinical development. Intellia Therapeutics has designed this treatment to knockout the *KLKB1* gene—encoding plasma PK—using CRISPR/Cas9 technology. Hereby *NTLA-2002*® aims to prevent the production of prekallikrein long-term through a single course of treatment [59].

*Berotralstat*, the first approved oral PKa inhibitor for LTP, was investigated in liquid formulation for ODT in the ZENITH-1 study. This phase 2 trial showed that a single dose of 750 mg reduced symptom severity and use of additional rescue medication, with a favourable safety profile [60]. Three other *oral PKa inhibito*rs under development are *ATN-249*®, *KVD824*®, and *KVD900*® [61, 62]. *ATN-249*®, developed for LTP, has recently successfully completed a phase 1 trial [63]. The results of the phase 2 trial of *KVD900*®, investigating its effectiveness as ODT, are expected in 2021 [64]. *KVD824*® has a markedly longer half-life than *KVD900*®, and therefore, its intended use is LTP. *KVD824*® has successfully completed its first-in-human study and a phase 2 trial is expected to start soon [65].

*PHA022121*® is the first oral drug (in the form of soft gel capsules) targeted at the BKRB2 receptor for use in acute attacks of HAE (ODT). This is a selective, competitive receptor antagonist that was shown to be highly potent in preclinical studies [66]. The phase 1 trial started in July 2019 and a phase 2 trial is expected to start soon.

Ambitiously, the first *gene therap*y strategies are already in preclinical development. An *adeno-associated virus-mediated antibody delivery gene therapy* demonstrated the induction of sustained and adequate plasma levels of C1-INH for at least 24 weeks in a murine model of HAE, by introducing an extrachromosomal copy of *SERPING1* into the cells [67]. BioMarin (*BMN 331*®) [68] and Regenxbio [69] are currently conducting preclinical experiments with these novel adeno-associated virus gene therapies for HAE. The potential long-term protection after a single treatment with such therapies is promising. Naturally, clinical safety and efficacy studies will be necessary before any further conclusions about these new therapeutic strategies can be drawn. Another challenge is the high levels of functional C1-INH levels required to effectively reduce breakthrough attacks [70]. The residual functional C1-INH in most C1-INH-HAE patients is 10–15%, while the minimal amount of C1-INH required to effectively prevent attacks is above 38% [71]. This contrasts to congenital factor IX deficiency (haemophilia B) for instance, in which only small antigenic increases of factor IX by gene therapy can induce marked reductions in disease severity [72].

## Discussion

This article focused on the current and future therapeutic options for C1-INH-HAE. With the development of highly effective therapies that are more easily administered and cause fewer side-effects than the conventional drugs, treatment decisions for C1-INH-HAE are expected to shift towards more personalized preferences with better disease control, provided that these drugs are affordable and accessible.

Within the last few years, two new LTP options became available and tremendously changed the field of C1-INH-HAE treatment. These options concern the *subcutaneous *C1-INH* concentrate* and the *PKa inhibitor lanadelumab*. Both are highly effective in the prevention of breakthrough attacks and showed impressive results, with high proportions of patients experiencing a complete lack of HAE attacks in their respective trials.

The present shift in treatment options will raise new clinical questions. Although the International/Canadian HAE guideline recommends both *subcutaneous *C1-INH or *lanadelumab* as first-line therapy [29], adherence to this recommendation may become restricted by the high costs of treatment. It will therefore be essential to determine the indications and identify patients that may benefit the most from this newest generation of treatments. Furthermore, it is currently unknown whether STP prior to invasive procedures remains necessary in patients with well controlled HAE under highly effective LTP. Moreover, once patients require parenteral STP or ODT less often, they might lose their skills to self-administer these drugs. Thus, better disease control could seemingly adversely lead to more emergency department visits by C1-INH-HAE patients, when they require intravenous agents for ODT. In addition, orally administered ODT may pose other drawbacks, such as impaired swallowing during laryngeal or oropharyngeal angioedema attacks, or abdominal attacks (due to vomiting), and a potentially reduced absorption during abdominal attacks. In the latter case, oral drugs may work less rapid than parenteral administered therapies. Nonetheless, oral ODT would certainly shorten the time to administration in patients who are currently dependent on healthcare professionals for the administration of parenteral ODT and in patients with reluctance to injections.

Another potential unexplored issue concerns the concomitant use of both ODT and LTP with the same mechanism of action. Whether *PKa inhibitors* developed for ODT will be effective in patients on LTP targeting the same protein remains to be elucidated. Moreover, it is still uncertain whether current limitations for C1-INH-HAE patients, such as the avoidance of *estrogen-containing contraceptives*, will remain necessary.

Even though the therapeutic options have widely expanded, the number of treatment options remains limited in certain subgroups of patients. For pregnant or breastfeeding patients, as well as children under the age of 12 years, the licensed therapies were virtually unchanged in recent years. This review focused on patients with C1-INH-HAE, since the aforementioned drugs have been primarily investigated in this population. Whether their clinical efficacy is similar in patients with other BK-mediated angioedema (such as nC1-INH-HAE or angiotensin-converting enzyme inhibitor-induced angioedema) needs to be assessed in future trials. Thinking further ahead, the first steps have been made in the domain of *gene therapy* for C1-INH-HAE. This opens the field for completely novel treatment options.

## Conclusion

Increased insights in the pathophysiology of HAE have paved the way for the development of new and more targeted therapies. The availability of more easily administered ODT options has improved both patients’ safety and quality of life. The anticipated shift towards highly effective, less frequently administered, and easily used LTP will hopefully further reduce the frequency and severity of HAE attacks and ameliorate the burden on HAE patients.

## Abbreviations

AA: Attenuated androgens
BK: Bradykinin
BKRB2: Bradykinin receptor B2
C1-INH: C1 esterase inhibitor
C1-INH-HAE: HAE due to C1-INH deficiency
EMA: European Medicines Agency
FDA: Food and Drug Administration
FFP: Fresh frozen plasma
FXII: Factor XII
FXIIa: Activated factor XII
HAE: Hereditary angioedema
HK: High-molecular-weight kininogen
KKS: Kallikrein/kinin system
LTP: Long-term prophylaxis
MASP: Mannose-binding lectin-associated serine protease
nC1-INH-HAE: HAE with normal C1-INH
ODT: On demand therapy
PKa: Plasma kallikrein
QoL: Quality of life
SERPIN: Serine protease inhibitor
SiRNA: Small interfering RNA
STP: Short-term prophylaxis
